# Payer leverage and hospital compliance with a benchmark: a population-based observational study

**DOI:** 10.1186/1472-6963-7-112

**Published:** 2007-07-18

**Authors:** John M Hollingsworth, Sarah L Krein, David C Miller, Sonya DeMonner, Brent K Hollenbeck

**Affiliations:** 1 Department of Urology, University of Michigan, 1500 East Medical Center Drive, 3876 Taubman Center, Ann Arbor, Michigan 48109-0330, USA; 2 VA HSR&D, VA Ann Arbor HCS, 2215 Fuller Road, Ann Arbor, Michigan 48113-0170, USA

## Abstract

**Background:**

Since 1976, Medicare has linked reimbursement for hospitals performing organ transplants to the attainment of certain benchmarks, including transplant volume. While Medicare is a stakeholder in all transplant services, its role in renal transplantation is likely greater, given its coverage of end-stage renal disease. Thus, Medicare's transplant experience allows us to examine the role of payer leverage in motivating hospital benchmark compliance.

**Methods:**

Nationally representative discharge data for kidney (*n *= 29,272), liver (*n *= 7,988), heart (*n *= 3,530), and lung (*n *= 1,880) transplants from the Nationwide Inpatient Sample (1993 – 2003) were employed. Logistic regression techniques with robust variance estimators were used to examine the relationship between hospital volume compliance and Medicare market share; generalized estimating equations were used to explore the association between patient-level operative mortality and hospital volume compliance.

**Results:**

Medicare's transplant market share varied by organ [57%, 28%, 27%, and 18% for kidney, lung, heart, and liver transplants, respectively (*P *< 0.001)]. Volume-based benchmark compliance varied by transplant type [85%, 75%, 44%, and 39% for kidney, liver, heart, and lung transplants, respectively (*P *< 0.001)], despite a lower odds of operative mortality at compliant hospitals. Adjusting for organ supply, high market leverage was independently associated with compliance at hospitals transplanting kidneys (OR, 143.00; 95% CI, 18.53 – 1103.49), hearts (OR, 2.84; 95% CI, 1.51 – 5.34), and lungs (OR, 3.24; 95% CI, 1.57 – 6.67).

**Conclusion:**

These data highlight the influence of payer leverage–an important contextual factor in value-based purchasing initiatives. For uncommon diagnoses, these data suggest that at least 30% of a provider's patients might need to be "at risk" for an incentive to motivate compliance.

## Background

Recently, an article in the *Los Angeles Times *cited that "about a fifth of federally funded (heart, liver, and lung) transplant programs fail to meet the government's minimum standards" and "perform too few operations to ensure competency" [1]. After being picked up by the Associated Press and several other major media outlets, the *Times' *article garnered the attention of Senator Charles Grassley (Chairman of the Senate Finance Committee), prompting him to begin a federal inquiry. Senator Grassley "has ordered investigations (and) demanded answers after ... reports detailing dangerous lapses in oversight of the national transplantation system." [2]. In response, Dr. Mark McClellan, a former senior administrator for the Centers for Medicare and Medicaid Services, has spoken out in defence of Medicare's transplant monitoring program [3]. Currently, empirical data addressing the concerns germane to this emerging policy debate are lacking.

Medicare's "minimum standards" for transplant programs, and issues surrounding hospital compliance with these benchmarks, have been in existence for thirty years. Since 1976, hospitals performing kidney transplants under the Medicare program have been required to meet benchmarks for accreditation, including kidney transplant volume [4]. With its successive coverage announcements for heart, liver, and lung transplants [5-7], Medicare has mandated similar volume standards, thereby linking benchmarking to reimbursement for all major solid organ transplants. However, as the *Times' *report illustrates, hospital compliance with these benchmarks varies widely [1]. Even prior to the recent media attention, concerns have been raised regarding the appropriateness of Medicare's standards and a possible lack of rigorous transplant program supervision [8]. These factors may explain, in part, the observed low rates of hospital compliance. Another important contextual factor may be Medicare's leverage with hospitals as a payer of transplantation services to promote compliance (i.e., payer market leverage).

Under the 1972 Social Security Act Amendments, Medicare guaranteed coverage to all patients with chronic renal failure requiring hemodialysis [9]. These benefits extend to kidney transplant services, as well. Thus, Medicare's stake in kidney transplantation is significant. However, no such entitlement presently exists for patients with other chronic disease states that commonly lead to organ transplantation; therefore, Medicare's payer market leverage is expected to be lower for other organ sites.

For this reason, we examined hospital transplant program compliance with Medicare's volume-based criteria, as well as the relationship between Medicare's market leverage and hospital benchmark compliance using national discharge data. Because payer market leverage is a likely determinant of the effectiveness of contemporary pay-for-performance programs [10-12], the Medicare experience has implications beyond the current debate surrounding transplant programs. As the ability of value-based purchasing initiatives to improve healthcare quality relies on provider compliance, this study provides empirical data that characterize the relationship between leverage and compliance.

## Methods

### Data source

Data from the Healthcare Cost and Utilization Project's Nationwide Inpatient Sample (NIS) between years 1993 and 2003 were used in this analysis. The NIS is a 20% stratified sample of all hospital discharges in the U.S. and consists of uniform hospital discharge summaries from non-federal acute-care hospitals throughout the U.S. [13]. It is a useful dataset for multi-year longitudinal studies. To date, more than 25 manuscripts have been published using multiple years of the NIS that cover a breadth of healthcare topics, including solid organ transplantation [14-17]. In accordance with the Code of Federal Regulations Title 45 Subpart A Section 46.101 paragraph b subparagraph 4, Institutional Review Board approval was waived for this study.

### Subjects

Using *International Classification of Disease, 9^th ^Revision, Clinical Modification *(ICD-9) procedure codes, we identified patients undergoing kidney (55.6, 55.69), liver (50.5, 50.51, 50.59), heart (33.6, 37.5), lung (33.5, 33.50, 33.51, 33.52), and combined heart-lung (33.6) transplants. Because Medicare has separate participation requirements for pediatric transplant programs, subjects under 18 years of age were excluded. Based upon this sampling methodology, a cohort was identified consisting of 29,272 kidney, 7,988 liver, 3,530 heart, and 1,880 lung transplants that were performed at 173, 88, 100, and 66 hospitals, respectively. Data on patient demographics (age, gender, race/ethnicity, admission acuity, diagnosis codes and primary payer) and hospital characteristics (teaching, profit, and proprietary status, urban/rural location, geographic census region, and hospital bed capacity) were also abstracted from the NIS. Secondary ICD-9 diagnosis codes were used to identify a set of 30 comorbidities as described by Elixhauser [18].

After aggregating data on each transplant recipient's primary payer to the hospital-level, a binary variable was constructed, identifying those hospitals performing transplants with a high proportion of Medicare patients. The literature suggests that an incentive applying to less than 15% to 20% of a provider's patients is unlikely to alter provider behavior [11]; therefore, an indicator variable was constructed to identify hospitals where 20% of transplants, by type, were reimbursed through Medicare and thereby distinguish those hospitals in which Medicare had low vs. high market leverage.

To estimate the supply of available donor organs, each hospital performing transplants was assigned to one of the 11 U.S. regions created by the United Network for Organ Sharing (UNOS), based on the hospital's two-letter state postal code as provided by the NIS. Data from UNOS on the number of donors recovered in the U.S. by organ type [19] were then merged with the study population files on UNOS region and year.

### Ascertainment of hospital volume compliance

The number of transplant procedures performed at each hospital was ascertained using a unique hospital identification code. Based on Medicare's coverage announcement for lung transplants [7], combined heart-lung transplants were counted toward both the heart and lung case volume totals for an individual hospital. Each hospital was assigned a volume for each year it participated. Each individual hospital-year was considered independently. Binary variables were then constructed indicating whether the hospital-year was compliant with Medicare's volume benchmark for each transplant type. The hospital-year volumes and binary volume compliance variables were then assigned to the discharge records that emanated from them. This was done for each transplant type, using those minimum volume requirements specified in the *Federal Register*. Specifically, hospitals were required to perform a minimum of 15, 12, 12, and 10 kidney, liver, heart, and lung transplants, respectively, over a 12-month period for Medicare approval [4-7].

### Outcomes

The primary outcomes for this study were the proportion of hospitals performing transplants compliant with Medicare's volume criteria and patient-level operative mortality, defined as death prior to hospital discharge.

### Statistical analyses

With the hospital-year as the unit of analysis, bivariate comparisons were performed for volume compliant and non-compliant hospitals based on transplant procedure strata. These comparisons focused on hospital structural attributes, as well as the clinical characteristics of their patients. T-tests were performed for continuous variables, and chi-square tests were performed for categorical variables.

The relationship between hospital volume compliance and Medicare market share was examined through logistic regression techniques with the use of robust variance estimates to adjust for potential cluster effects [20]. The concern for cluster effects arose because of the potential for a hospital to be included in a multiple number of years, which could induce a correlation and would require adjustment.

For these models, the unit of analysis was the hospital-year. The outcome was CMS volume benchmark compliance. The exposure was high Medicare market leverage (the binary variable described above). Given the well-documented regional variation in transplantation [21], these models were adjusted for donor organ supply, introduced as a continuous count. The count of donor organ supply was UNOS-region specific, into which each hospital was sorted. These models were also adjusted for hospital case mix. For this, patient-level data were aggregated to the hospital level in order to derive a mean count of the Elixhauser codes [18] specific for the four different transplant procedures, which described each hospital's "typical" patient.

Medicare's coverage announcement for lung transplants was made in 1995 [7]. This allowed for a secondary analysis, in which the odds of volume compliance as a function of Medicare's market share were compared prior to (1993 to 1994) and after (1995 to 2003) the specification of minimum caseloads.

Next, the relationship between patient-level operative mortality and hospital volume compliance was examined using generalized estimating equations (GEE). Given that not all of the hospitals were represented in multiple years of our sampling frame, the data did not represent a true panel structure. However, there was potential correlation between observations as patients were clustered within hospitals. Therefore, GEE models with a logit link and an exchangeable correlation matrix were fitted to produce a population-averaged odds ratio (OR) and a robust estimator to correct the standard errors for the potential correlation of observations within hospitals [22].

For our analysis of operative mortality (outcome), our unit of analysis was at the patient level. Our exposure was hospital-level volume benchmark compliance. Each model was adjusted for patient age, gender, race, comorbidity, and insurance type, as well as treatment year, teaching status, for-profit status, ownership, hospital bed capacity and census region. Case-mix adjustment was accomplished using a subset of the Elixhauser comorbidity flags [18], in which those comorbidities directly related to the principal diagnosis were removed.

In addition to the analysis using these basic GEE models, sensitivity analyses were conducted to test the potential affect of several assumptions. In particular, hospital participation in the NIS varies by year with some hospitals included in multiple years. Specifically, 111, 54, 60, and 40 hospitals performing kidney, liver, heart, and lung transplants were sampled in multiple years. To determine whether those with more frequent participation had an undue influence on the results, a dataset was constructed that included a randomly selected year of data only for those hospitals that were sampled multiple times as part of the NIS sampling frame. The operative mortality analyses were then repeated using this new dataset. Since the results remained consistent across these analyses, only those results from the primary GEE models are reported. All statistical analyses were two-tailed, with a 5% significance level, and were conducted using Stata version 9.0 (Stata Corp, College Station, Texas).

## Results

**Figure 1:**
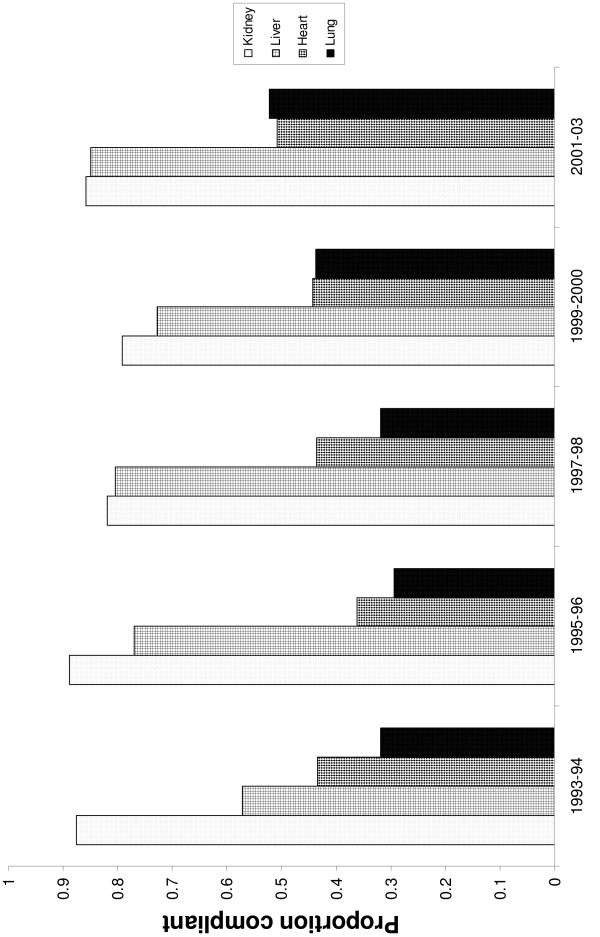
Transplant center compliance with Medicare's volume criteria stratified by year.

Over the study interval, Medicare was the primary payer for 57% of kidney, 28% of lung, 27% of heart, and 18% of liver transplants (*P *< 0.001). The proportion of hospitals compliant with Medicare's volume benchmarks varied by transplant type, ranging from as low as 24% of hospitals performing lung transplants in 1995 to as high as 96% of hospitals performing kidney transplants in 2003. Average compliance for hospitals performing kidney transplants was 85%, 75% for liver transplants, 44% for heart transplants, and 39% for lung transplants (*P *< 0.001). Temporal changes in volume compliance status stratified by transplant type are depicted in Figure 1. Patients receiving kidney transplants at volume compliant hospitals were generally older, and a higher proportion were male when compared to those treated at non-compliant hospitals (Table 1). A higher proportion of volume compliant kidney and liver transplant hospitals were teaching facilities with a larger bed capacity than non-compliant hospitals (Table 2).

**Table 1:** Comparisons between the average transplant recipient at Medicare volume compliant and non-compliant hospitals.^†^

	KIDNEY			LIVER			HEART			LUNG		
	Non-compliant (*n *= 40)	Compliant (*n *= 133)	*P*-value	Non-compliant (*n *= 29)	Compliant (*n *= 59)	*P*-value	Non-compliant (*n *= 60)	Compliant (*n *= 40)	*P*-value	Non-compliant (*n *= 40)	Compliant (*n *= 26)	*P*-value

Mean age ± std dev (years)	37.0 ± 12.9	45.2 ± 3.1	**	46.3 ± 14.1	49.4 ± 2.2		49.8 ± 11.1	51.4 ± 3.2		46.8 ± 13.6	49.4 ± 4.0	
Prop female	46%	41%	*	38%	38%		30%	24%	*	50%	50%	
Prop w/>15% Black	36%	37%		8%	8%		15%	21%		13%	3%	*
Prop elect admit	40%	38%		31%	22%	*	22%	22%		21%	16%	
Prop w/>20% Med pen	73%	100%	**	24%	38%		47%	71%	**	43%	71%	**

Abbreviations: std dev, standard deviation; prop, proportion; w/, with; elect admit, elective admission; Med pen, Medicare penetration

† T-tests assuming unequal variances were performed for continuous variables and chi-square analyses were performed for categorical variables.

*For *P *≤ 0.05.

**For *P *≤ 0.001.

**Table 2:** Comparisons between measures of structure for Medicare volume compliant and non-compliant hospitals.^†^

	Kidney			Liver			Heart			Lung		
	Non-compliant (*n *= 40)	Compliant (*n *= 133)	*P*-value	Non-compliant (*n *= 29)	Compliant (*n *= 59)	*P*-value	Non-compliant (*n *= 60)	Compliant (*n *= 40)	*P*-value	Non-compliant (*n *= 40)	Compliant (*n *= 26)	*P*-value

Prop teach	60%	84%	**	71%	88%	*	83%	87%		84%	90%	
Prop priv	45%	36%		48%	20%	**	42%	23%	**	35%	13%	*
Prop for-profit	4%	3%		5%	0%	*	3%	0%		3%	0%	
Prop urban	97%	100%	*	100%	100%		97%	100%	*	99%	100%	
Bedsize												
Small	19%	4.%	**	14%	4%	*	6%	1%		9%	0%	
Med	21%	20%		22%	16%		21%	18%		19%	24%	
Large	60%	76%		64%	80%		73%	81%		72%	76%	
Region												
NE	19%	25%		24%	16%		13%	11%		15%	8%	
NC	25%	23%		20%	29%		33%	28%		35%	27%	
South	33%	27%		29%	30%		33%	37%		33%	29%	
West	23%	25%		27%	25%		21%	24%		17%	36%	

Abbreviations: prop, proportion; teach, teaching; priv, private; med, medium; NE, northeast; NC, north-central

†Chi-square tests were performed for all categorical variables.

*For *P *≤ 0.05.

**For *P *≤ 0.001.

Further, high Medicare market leverage was found to be independently associated with hospital volume compliance for hospitals performing kidney [OR, 143.00; 95% confidence interval (CI), 18.53–1103.49], heart (OR, 2.84; 95% CI, 1.51 – 5.34), and lung (OR, 3.24; 95% CI, 1.57 – 6.67) transplants. A similar association, although not statistically significant (OR, 1.87; 95% CI, 0.87 – 4.04), was found for hospitals performing liver transplants. For those years of the study interval (1993 to 1994) during which no lung transplant minimum caseloads were specified, the OR for volume compliance was 1.38 (95% CI, 0.15 – 12.66). Following Medicare's coverage announcement, the OR rose to 3.73 (95% CI, 1.70 – 8.19).

Unadjusted operative mortality rates were 1% for kidney, 9% for liver, 9% for heart, and 11% for lung transplants. Results from the adjusted models are presented in Tables 3, 4, 5, 6. Patients undergoing liver transplantation at non-compliant hospitals had a significantly greater risk of operative mortality (OR, 1.50; 95% CI, 1.12 – 2.02), even after adjustment for age, gender, race, comorbidity status, treatment year, and insurance type. A trend toward greater operative mortality risk was also observed for non-compliant hospitals performing kidney, heart, and lung transplantation, although these results did not reach statistical significance.

**Table 3:** Adjusted odds of operative mortality following kidney transplantation at volume non-compliant hospitals.

COvariate	Odds Ratio	95% Confidence Interval
Volume non-compliant	1.64	0.77 – 3.52
Female	1.01	0.80 – 1.26
Race		
White	1 (ref.)	--
Black	1.05	0.76 – 1.45
Hispanic	0.94	0.60 – 1.48
Asian	1.21	0.70 – 2.07
Other	1.05	0.72 – 1.51
Insurance type		
Medicare	1 (ref.)	--
Medicaid	0.50	0.38 – 0.66
Private	1.42	0.96 – 2.10
Operative year		
1993	1 (ref.)	--
1994	1.21	0.71 – 2.07
1995	1.03	0.59 – 1.77
1996	1.35	0.80 – 2.27
1997	1.21	0.72 – 2.04
1998	0.61	0.31 – 1.19
1999	0.73	0.40 – 1.36
2000	0.57	0.29 – 1.10
2001	0.82	0.43 – 1.56
2002	0.54	0.27 – 1.07
2003	0.81	0.44 – 1.48
Count of Elixhauser codes	1.30	1.20 – 1.41
Hospital census region		
Northeast	1 (ref.)	--
North-central	0.74	0.46 – 1.17
South	0.89	0.59 – 1.35
West	0.60	0.38 – 0.94
Hospital bedsize		
Small	1 (ref.)	--
Medium	0.93	0.44 – 1.96
Large	0.96	0.47 – 1.93
For-profit	1.47	0.50 – 4.32
Private	0.82	0.55 – 1.22
Teaching	1.12	0.69 – 1.82

**Table 4:** Adjusted odds of operative mortality following liver transplantation at volume non-compliant hospitals.

COvariate	Odds Ratio	95% Confidence Interval
Volume non-compliant	1.50	1.12 – 2.02
Female	1.16	1.02 – 1.33
Race		
White	1 (ref.)	--
Black	0.94	0.58 – 1.53
Hispanic	1.13	0.83 – 1.55
Asian	1.01	0.67 – 1.51
Other	1.28	0.99 – 1.66
Insurance type		
Medicare	1 (ref.)	--
Medicaid	0.81	0.64 – 1.02
Private	0.85	0.66 – 1.08
Operative year		
1993	1 (ref.)	--
1994	0.80	0.49 – 1.31
1995	0.70	0.41 – 1.19
1996	0.75	0.43 – 1.29
1997	0.62	0.34 – 1.13
1998	0.44	0.25 – 0.77
1999	0.66	0.44 – 0.98
2000	0.52	0.33 – 0.81
2001	0.42	0.23 – 0.74
2002	0.41	0.26 – 0.64
2003	0.47	0.30 – 0.74
Elixhauser codes		
Congestive heart failure	2.54	1.63 – 3.94
Arrhythmias	2.08	1.47 – 2.93
Valvular disease	0.61	0.32 – 1.17
Pulmonary circulation disorders	3.48	2.09 – 5.80
Peripheral vascular disorders	1.21	0.44 – 3.28
Paralysis	2.27	0.62 – 8.22
Neurologic disorders	1.09	0.52 – 2.27
Chronic pulmonary disease	1.04	0.67 – 1.60
Diabetes mellitus, complex	0.94	0.54 – 1.63
Hypothyroidism	0.54	0.28 – 1.04
Renal failure	1.34	1.02 – 1.77
Peptic ulcer disease	0.62	0.28 – 1.38
Rheumatoid arthritis	0.98	0.38 – 2.50
Coagulopathy	1.86	1.55 – 2.23
Weight loss	0.71	0.52 – 0.99
Fluid and electrolyte disorders	1.23	1.02 – 1.49
Blood loss anemia	1.21	0.59 – 2.50
Deficiency anemias	0.59	0.38 – 0.93
Psychoses	0.32	0.10 – 1.00
Depression	0.17	0.04 – 0.67
Hospital census region		
Northeast	1 (ref.)	--
North-central	0.45	0.34 – 0.61
South	0.61	0.50 – 0.75
West	0.53	0.40 – 0.71
Hospital bedsize		
Small	1 (ref.)	--
Medium	0.66	0.46 – 0.95
Large	0.79	0.58 – 1.08
For-profit	1.18	0.06 – 21.83
Private	0.96	0.75 – 1.23
Teaching	1.04	0.78 – 1.38

**Table 5:** Adjusted odds of operative mortality following heart transplantation at volume non-compliant hospitals.

COvariate	Odds Ratio	95% Confidence Interval
Volume non-compliant	1.19	0.92 – 1.54
Female	1.57	1.23 – 2.00
Black	0.88	0.58 – 1.36
Insurance type		
Medicare	1 (ref.)	--
Medicaid	0.77	0.61 – 0.98
Private	0.58	0.40 – 0.86
Operative year		
1993	1 (ref.)	--
1994	1.15	0.72 – 1.85
1995	0.61	0.32 – 1.19
1996	0.78	0.44 – 1.39
1997	0.75	0.48 – 1.17
1998	0.51	0.28 – 0.95
1999	1.18	0.72 – 1.92
2000	0.86	0.49 – 1.52
2001	0.51	0.27 – 0.96
2002	0.73	0.38 – 1.38
2003	0.63	0.34 – 1.16
Elixhauser codes		
Diabetes mellitus, complex	0.54	0.18 – 1.68
Renal failure	1.09	0.59 – 1.98
Liver disease	6.05	2.04 – 18.00
Peripheral vascular disorders	1.43	0.75 – 2.71
Paralysis	1.49	0.31 – 7.08
Neurologic disorders	2.78	1.74 – 4.44
Chronic pulmonary disease	0.66	0.41 – 1.08
Hypothyroidism	0.61	0.29 – 1.30
Rheumatoid arthritis	0.51	0.06 – 4.50
Coagulopathy	3.23	2.48 – 4.21
Weight loss	1.25	0.55 – 2.87
Fluid and electrolyte disorders	1.03	0.80 – 1.32
Blood loss anemia	0.59	0.14 – 2.55
Deficiency anemias	0.09	0.02 – 0.39
Psychoses	0.43	0.05 – 3.37
Depression	0.35	0.08 – 1.52
Hospital census region		
Northeast	1 (ref.)	--
North-central	0.48	0.34 – 0.67
South	0.43	0.31 – 0.60
West	0.37	0.25 – 0.56
Hospital bedsize		
Small	1 (ref.)	--
Medium	0.50	0.19 – 1.28
Large	0.49	0.21 – 1.15
For-profit	6.26	0.33 – 119.38
Private	1.13	0.85 – 1.50
Teaching	0.97	0.69 – 1.35

**Table 6:** Adjusted odds of operative mortality following lung transplantation at volume non-compliant hospitals.

COvariate	Odds Ratio	95% Confidence Interval
Volume non-compliant	1.43	0.83 – 2.46
Female	0.95	0.75 – 1.20
Black	1.45	0.72 – 2.89
Insurance type		
Medicare	1 (ref.)	--
Medicaid	0.83	0.59 – 1.16
Private	0.78	0.47 – 1.29
Operative year		
1993	1 (ref.)	--
1994	0.81	0.38 – 1.71
1995	0.65	0.27 – 1.55
1996	1.26	0.60 – 2.61
1997	1.03	0.46 – 2.28
1998	0.49	0.15 – 1.58
1999	1.03	0.46 – 2.32
2000	0.95	0.29 – 3.17
2001	0.56	0.23 – 1.33
2002	1.04	0.42 – 2.60
2003	0.67	0.28 – 1.58
Count of Elixhauser codes	0.98	0.86 – 1.12
Hospital census region		
Northeast	1 (ref.)	--
North-central	0.67	0.39 – 1.16
South	0.87	0.50 – 1.53
West	0.83	0.47 – 1.47
Hospital bedsize		
Small	1 (ref.)	--
Medium	0.55	0.15 – 2.01
Large	0.42	0.13 – 1.38
For-profit	1.17e-12	1.73e-13 – 7.93e-12
Private	0.86	0.47 – 1.55
Teaching	0.73	0.35 – 1.52

## Discussion

Consistent with lay press reports, we have demonstrated substantial hospital non-compliance with Medicare's volume-based benchmarks for heart and lung transplant programs. We found that benchmark compliance varies by transplant type despite the fact that patients undergoing liver transplantation at volume compliant hospitals have lower odds of operative mortality. Importantly, benchmark compliance was observed to vary with Medicare's market share for each organ type, with compliance being greatest among hospitals performing kidney transplants, where Medicare was the primary payer for 57% of discharges. In contrast, benchmark compliance was lowest for lung transplantation, a procedure where Medicare was the primary payer for only 28% of cases.

Medicare's policy for transplant programs is predicated on the assumption that hospital compliance with volume standards will translate into better patient outcomes. Along this line of thinking, Medicare has linked both transplant program accreditation and procedural reimbursement to the attainment of its benchmarks. While Medicare's volume thresholds were initially set without any evidence base, substantial literature has since been published documenting higher mortality after selected high risk procedures, including organ transplantation, at low-volume centers [23-27]. Despite this, these data suggest that even the combination of a valid benchmark and a powerful incentive (accreditation and reimbursement in this case) may be insufficient to ensure uniform hospital/provider compliance.

Accordingly, these data highlight the importance of other contextual factors (e.g., payer leverage) on the success of performance-based incentive programs. Efforts to motivate providers through financial incentives are thought to have a limited effect unless the payer promoting the incentive program represents a substantial proportion of the provider's practice [11,12]. It is estimated that an incentive program applying to less than 15% to 20% of a provider's patient panel is unlikely to induce provider response [11]. For infrequent diagnoses and procedures–heart and lung transplants in this study–these data suggest that such leverage may be inadequate to motivate change.

These findings must be considered in the context of several limitations. To begin, donor organs represent a scarce commodity with a relatively fixed supply and an inelastic demand [28]. As such, concerns surround not only the attainability of Medicare's volume benchmarks, but also Medicare's ability to leverage its market share to affect hospital compliance. With an estimated 14,000 kidney, 5,000 liver, 2,000 heart, and 1,000 lung transplants performed annually [29] at 244 kidney, 123 liver, 135 heart, and 68 lung transplant centers nationwide [19], Medicare's volume thresholds would appear reasonable if the distribution of donor organ supply was equitable across centers.

Second, in this study, high Medicare market share was independently associated with higher odds of compliance, even after adjustment for regional donor organ supply. However, our adjustment for supply alone may be imperfect, as compliance among hospitals performing liver transplantation, for which Medicare has the smallest market share, was similar to that of kidney transplantation rather than heart or lung transplantation. This indicates that there may be other factors influencing the caseload size for hospitals (e.g., the number of patients with end stage renal disease in the nearby area). Nevertheless, this study highlights the interplay between market share and supply as potential determinants of compliance with volume-based benchmarks.

A third limitation concerns this study's observational design. Given the inherent endogeneity of the data, the directionality of the association between Medicare market share and hospital compliance cannot be directly discerned. For example, what we have interpreted as a leverage effect may instead be a selection effect. However, these data do suggest a trend towards increasing compliance across transplant types following Medicare's successive transplant coverage decisions (Figure 1) [4-7]. Further, we are reassured by the results from our secondary analysis in which we examined the relationship between compliance and Medicare market share for lung transplants in the absence of a minimum threshold.

Fourth, transplant-relevant clinical endpoints are limited within these administrative data. While alternative transplant outcomes (e.g., incidence of delayed graft function and graft survival) would have made this analysis richer, we could only examine the clinical and resource use information included in a typical discharge abstract. Moreover, our primary measure, operative mortality, is an infrequent event, particularly following kidney transplant [30]. Though a significant difference in patient mortality for liver transplantation was demonstrated at volume compliant vs. non-compliant hospitals, the ability to detect a difference for kidney, heart, and lung transplants was perhaps limited by sample size. Further investigation is, therefore, warranted to examine the effect of volume compliance on more common, long-term outcomes using clinical data.

Finally, comment can only be made on a hospital's attainment of Medicare's transplant volume benchmarks. A hospital's actual Medicare accreditation status cannot be ascertained within the NIS. Medicare requires that transplant programs undergo review every three years; however, even prior to the recent *Los Angeles Times *report, the Office of the Inspector General raised questions about the rigor of this oversight [8]. A new rule change has been proposed to address these concerns [31], but current lack of enforcement may contribute to the relatively low compliance among heart and lung transplant programs.

## Conclusion

This study provides a nationally representative estimate of hospital compliance with Medicare's volume criteria for transplant programs and suggests the potential influence of payer market share in motivating benchmark compliance. While others have suggested that an incentive applying to less than 15% to 20% of a provider's patient panel is unlikely to induce provider response [10], these findings would indicate that for infrequent diagnoses and procedures, more than 30% of provider's patients might need to be impacted by an incentive in order to motivate change. These data also have implications for Medicare's current pay-for-performance initiatives [32]. For those public health issues that disproportionately affect patients under age 65, Medicare may lack the leverage to alter provider behavior. Given the fragmented nature of the U.S. healthcare system, multilateral efforts between Medicare and other payers may, therefore, be necessary to achieve the intended aims of specific value-based purchasing initiatives.

## Competing interests

The author(s) declare that they have no competing interests.

## Authors' contributions

JH participated in the this study's conception and design, the acquisition of data, the analysis and interpretation of data, drafting of the manuscript, critical revision of the manuscript, and all of the statistical analyses. SL participated in the analysis and interpretation of data, drafting of the manuscript, critical revision of the manuscript, all statistical analyses, and supervision of this study. DM participated in this study's conception and design, the interpretation of data, and critical revision of the manuscript. SD participated in the analysis and interpretation of data, critical revision of the manuscript, and all statistical analyses. BH participated in this study's conception and design, the acquisition of data, analysis and interpretation of data, drafting of the manuscript, critical revision of the manuscript, administrative and technical support, and supervision of this study.

## Pre-publication history

The pre-publication history for this paper can be accessed here:



## References

[B1] Weber T, Ornstein C 20% of U.S. transplant centers are found to be substandard. Los Angeles Times.

[B2] Weber T, Ornstein C Report on transplant programs prompts inquiry by U.S. Senator. Los Angeles Times.

[B3] Ornstein C Medicare's transplant monitoring defended. Los Angeles Times.

[B4] (1976). Conditions for ESRD coverage.

[B5] Medicare Program (1987). Criteria for Medicare coverage of heart transplants–HCFA. Notice of HCFA ruling. Fed Regist.

[B6] Medicare Program (1991). Criteria for Medicare coverage of adult liver transplants–HCFA. Final notice. Fed Regist.

[B7] Medicare Program (1995). Criteria for Medicare coverage of lung transplants–HCFA. Notice with comment period. Fed Regist.

[B8] Department of Health and Human Services (2005). Medicare approved heart transplant centers. OIG Report No OEI-01-02-00520.

[B9] Rettig RA (1980). Implementing the End-Stage Renal Disease Program of Medicare.

[B10] Dudley RA (2005). Pay-for-performance research: how to learn what clinicians and policy makers need to know. JAMA.

[B11] Epstein AM, Lee TH, Hamel MB (2004). Paying physicians for high-quality care. New Engl J Med.

[B12] Rosenthal MB, Fernandopulle R, Song HR, Landon B (2004). Paying for quality: providers' incentives for quality improvement. Health Aff (Millwood).

[B13] Houchens RL, Elixhauser A Using the HCUP nationwide inpatient sample to estimate trends. HCUP Methods Series Report # 2005-01.

[B14] Xiao H, Campbell ES, Song KS (2002). A trend analysis of organ transplantation among ethnic groups. J Natl Med Assoc.

[B15] Birmkeyer JD, Siewers AE, Finlayson EV (2002). Hospital volume and surgical mortality in the United States. N Engl J Med.

[B16] Ritchie JL, Maynard C, Chapko MK, Every NR, Martin DC (1999). Association between percutaneous transluminal coronary angioplasty volumes and outcomes in the Healthcare Cost and Utilization Project 1993–1994. Am J Cardiol.

[B17] Konety BR, Allareddy V, Modak S, Smith B (2006). Mortality after major surgery for urologic cancers in specialized urology hospitals: are they any better?. J Clin Oncol.

[B18] Elixhauser A, Steiner C, Harris DR, Coffey RM (1998). Comorbidity measures for use with administrative data. Med Care.

[B19] United Network for Organ Sharing Web site. http://www.unos.org.

[B20] Rogers WH (1992). Regression standard errors in clustered samples. Stata Technical Bulletin.

[B21] Ellison MD, Edwards LB, Edwards EB, Barker CF (2003). Geographic differences in access to transplantation in the United States. Transplantation.

[B22] Chao EC (2006). Structured correlation in models for clustered data. Stat Med.

[B23] Begg CB, Cramer LD, Hoskins WJ, Brennan MF (1998). Impact of hospital volume on operative mortality for major cancer surgery. JAMA.

[B24] Birkmeyer JD, Finlayson SR, Tosteson AN, Sharp SM, Warshaw AL, Fisher ES (1999). Effect of hospital volume on in-hospital mortality with pancreaticoduodenectomy. Surgery.

[B25] Axelrod DA, Guidinger MK, McCullough KP, Leichtman AB, Punch JD, Merion RM (2004). Association of center volume with outcome after liver and kidney transplantation. Amer J Transplant.

[B26] Edwards EB, Roberts JP, McBride MA, Schulak JA, Hunsicker LG (1999). The effect of the volume of procedures at transplantation centers on mortality after liver transplantation. New Engl J Med.

[B27] Hosenpud JD, Breen TJ, Edwards EB, Daily OP, Hunsicker LG (1994). The effect of transplant center volume on cardiac transplant outcome. A report of the United Network for Organ Sharing Scientific Registry. JAMA.

[B28] Ojo AO, Heinrichs D, Emond JC, McGowan JJ, Guidinger MK, Delmonico FL, Metzger RA (2004). Organ donation and utilization in the USA. Amer J Transplant.

[B29] Organdonor.gov Web site. http://www.organdonor.gov.

[B30] Scientific Registry of Transplant Recipients Web site. http://www.ustransplant.org.

[B31] (2005). Hospital conditions of participation: Requirements for approval and re-approval of transplant centers to perform organ transplants. 42 CFR Parts 405, 482, and 488 (proposed).

[B32] Centers for Medicare and Medicaid Services Web site. http://www.cms.hhs.gov/HospitalQualityInits/35_HospitalPremier.asp.

